# Cost-effectiveness analysis of toripalimab plus bevacizumab versus sorafenib as first-line treatment for advanced hepatocellular carcinoma in China

**DOI:** 10.3389/fimmu.2025.1676293

**Published:** 2025-12-09

**Authors:** Zhengxiong Li, Jing He, Kai Ma

**Affiliations:** 1 School of Medical Informatics and Engineering, Xuzhou Medical University, Xuzhou, China; 2 School of Medicine, University of Electronic Science and Technology of China, Chengdu, China

**Keywords:** cost-effectiveness, advanced hepatocellular carcinoma, toripalimab, bevacizumab, sorafenib, first-line, immunotherapy

## Abstract

**Background:**

The HEPATORCH trial demonstrated that toripalimab combined with bevacizumab significantly prolonged progression-free survival and overall survival in patients with advanced hepatocellular carcinoma (HCC), with an acceptable safety profile. However, its economic implications remain unclear. This study aimed to evaluate the cost-effectiveness of toripalimab plus bevacizumab versus sorafenib monotherapy as first-line treatment for advanced HCC from the Chinese healthcare system perspective.

**Methods:**

Using clinical data from the HEPATORCH trial, a three-state partitioned survival model was constructed to assess the cost-effectiveness of the two treatment strategies over a 10-year horizon. Cost and utility inputs were derived from the publicly available data and published literature. Primary outcomes included total costs, life-years (LYs), quality-adjusted life-years (QALYs), and incremental cost-effectiveness ratios (ICERs). Scenario analysis, one-way and probabilistic sensitivity analyses (PSA) were performed to evaluate model robustness.

**Result:**

Base-case analysis demonstrated that the toripalimab-bevacizumab combination and sorafenib monotherapy incurred total costs of $44,994.43 and $35,014.79, yielding 2.26 LYs and 1.79 LYs, and 1.57 QALYs and 1.16 QALYs, respectively. The toripalimab plus bevacizumab regimen provided an additional 0.41 QALYs at an incremental cost of $9,979.63, resulting in an ICER of $24,602.67/QALY. The ICER was significantly lower than the willingness-to-pay (WTP) threshold of three times China’s per capita gross domestic product ($40,334/QALY). Scenario analyses confirmed the robustness of the base-case results. One-way sensitivity analysis revealed that the cost of bevacizumab and the proportion of patients receiving subsequent therapy in the sorafenib group were the most influential parameters on the ICERs. PSA indicated a 95.76% probability of toripalimab combined with bevacizumab being cost-effective at the WTP threshold of $40,334 per QALY.

**Conclusion:**

Compared with sorafenib, toripalimab plus bevacizumab is likely a cost-effective first-line treatment option for advanced HCC in China.

## 1 Introduction

Liver cancer is the sixth most common cancer worldwide and the third leading cause of cancer-related death (1). Studies indicate that in 2020, China accounted for 45.3% of global new liver cancer cases, while the domestic 5-year survival rate was only 12.1% (2). As a major global health challenge, more than half of the cases and deaths occur in China (3, 4). Projections of the economic burden of liver cancer in China indicate a continued rise in societal costs in the coming years (4).

Approximately 70% to 80% of liver cancer cases are hepatocellular carcinoma (HCC) (5). Early-stage HCC is often clinically asymptomatic, and approximately 50% of patients are diagnosed at an advanced or even terminal stage, leaving systemic therapy as the only viable treatment option (6). The landscape of systemic treatment has evolved from single-agent targeted therapies to combination therapies involving immune checkpoint inhibitors plus targeted agents (7). However, only a small subset of patients achieves durable clinical benefits, underscoring the persistent therapeutic challenges in advanced HCC. Consequently, novel molecularly targeted monotherapy, new immuno-oncology monotherapy, and innovative combination therapies have emerged and demonstrated encouraging results in clinical trials (7). Notably, immune checkpoint inhibitors (ICIs) such as programmed death-1/programmed death-ligand 1 (PD-1/PD-L1) inhibitors have shown significant efficacy in improving overall survival (OS), but their use is limited due to the financial burden for individuals and governments (8).

Toripalimab is a humanized IgG4κ monoclonal antibody targeting PD-1, It binds to PD-1 and blocks its interaction with PD-L1 and PD-L2, thereby restoring T-cell-mediated antitumor activity. This agent first gained conditional approval in China in December 2018 for unresectable or metastatic melanoma after systemic treatment failure, marking its global debut. It has since been widely used in clinical practice for various malignancies including melanoma, nasopharyngeal carcinoma, esophageal squamous cell carcinoma, and non-small cell lung cancer (9, 10). A phase II study evaluating toripalimab plus bevacizumab as first-line therapy for advanced HCC showed promising efficacy and safety, with an objective response rate of 46.3% and median progression-free survival (PFS) of 9.8 months (95% confidence interval [CI]: 5.6 to not evaluable) (11). Subsequently, the phase III HEPATORCH trial demonstrated that toripalimab plus bevacizumab significantly improved OS compared with sorafenib (median 20.0 months [95% CI: 15.3-23.4] vs 14.5 months [95% CI: 11.4-18.8]; hazard ratio [HR]: 0.76, 95% CI: 0.58-0.99). The combination also significantly prolonged PFS (median 5.8 months [95% CI: 4.6-7.2] vs 4.0 months [2.8-4.2]; HR: 0.69, 95% CI: 0.53-0.91) (12). Based on these results, China’s National Medical Products Administration (NMPA) formally approved this regimen in March 2025 for first-line treatment of unresectable or metastatic HCC, marking the 11th approved indication for toripalimab in China (12).

While the combination of toripalimab and bevacizumab has shown survival benefits and favorable safety profile in patients with advanced HCC, a pharmacoeconomic evaluation for this treatment regimen is currently lacking. Given the absence of economic evidence supporting this regimen, this study aimed to assess the cost-effectiveness of toripalimab plus bevacizumab versus sorafenib as first-line therapy for advanced HCC from the Chinese healthcare system perspective. The findings might provide crucial evidence for guiding healthcare policy decisions, promoting rational drug use in clinical practice, and optimizing the allocation of limited medical resources.

## 2 Methods

As this economic evaluation used modeling techniques based solely on publicly available data and published literature, and did not involve human or animal subjects, ethics committee approval was not required. The analysis followed the Consolidated Health Economic Evaluation Reporting Standards 2022 (CHEERS 2022) guidelines (13), and the completed checklist is provided in **Supplementary Table S1**.

### 2.1 Patients and intervention

The study population characteristics were consistent with those in the HEPATORCH trial. Eligible patients were aged 18–75 years with histologically or cytologically confirmed HCC or HCC diagnosed per AASLD criteria in cirrhotic patients, had BCLC stage B or C disease not amenable to radical or locoregional therapy, had not received prior systemic therapy for advanced disease, and had Child-Pugh Class A liver function with ECOG performance status of 0 or 1. Key exclusion criteria included cholangiocarcinoma or mixed liver cancer, significant coagulation disorders, previous gastrointestinal bleeding within 6 months, and coinfection with HBV and HCV.

A total of 326 patients were randomly assigned (1:1) to receive either toripalimab (240 mg every 3 weeks for up to 35 cycles) plus bevacizumab (15 mg/kg, once every 3 weeks) or sorafenib (400 mg, twice daily). Treatment continued until disease progression, unacceptable toxicity, or other discontinuation criteria were met. After disease progression, patients received second-line therapies including immunotherapy (pembrolizumab), targeted therapy (regorafenib), or best supportive care (BSC). In the HEPATORCH trial, 92 patients (56.8%) in the toripalimab-bevacizumab group and 113 patients (68.9%) in the sorafenib group received subsequent systemic anticancer therapy (12), while the remaining patients received BSC. The proportions of patients receiving subsequent treatments in the two groups are detailed in **Table 1**.

**Table 1:** Model parameters inputs and the ranges of the sensitivity analysis.

Parameters	Baseline value	Range		Distribution	Reference
		Lower value	Upper value		
Clinical inputs					
OS survival mode for toripalimab group	meanlog = 2.9363 sdlog = 1.1358	NA	NA	Log-normal	Model fitting
PFS survival mode for toripalimab group	meanlog = 1.6667 sdlog = 1.1352	NA	NA	Log-normal	Model fitting
OS survival mode for sorafenib group	meanlog = 2.6781 sdlog = 1.0775	NA	NA	Log-normal	Model fitting
PFS survival mode for sorafenib group	meanlog = 1.3500 sdlog = 0.8960	NA	NA	Log-normal	Model fitting
Costs inputs (US $)					
Toripalimab (240 mg)	264.66	211.73	317.59	Gamma	(18)
Bevacizumab (100 mg)	155.02	134.38	210.62	Gamma	(18)
Sorafenib (200 mg)	3.38	0.70	12.54	Gamma	(18)
Regorafenib (40 mg)	7.62	0.59	24.22	Gamma	(18)
Pembrolizumab (100 mg)	2,515.97	2,012.78	3,019.16	Gamma	(18)
Best supportive care per cycle	366.63	293.30	439.96	Gamma	(25)
Follow-up and monitoring per cycle in PFS	83.13	66.50	99.76	Gamma	(22)
Follow-up and monitoring per cycle in PD	153.05	122.44	183.66	Gamma	(22)
Drug administration per unit	17.69	14.15	21.23	Gamma	(22)
End-of-life care	1,914.99	1,531.99	2,297.99	Gamma	(22)
Cost of AE per unit (US $)					
Anaemia	605.33	484.26	726.40	Gamma	(20)
Hypertension	35.53	28.42	42.64	Gamma	(21)
Proteinuria	106.29	85.03	127.55	Gamma	(21)
Thrombocytopenia	3,434.29	2,747.43	4,121.15	Gamma	(22)
Aspartate aminotransferase increased	180.02	144.02	216.02	Gamma	(24)
Blood bilirubin increased	107.70	86.16	129.24	Gamma	(23)
Alanine aminotransferase increased	89.02	71.22	106.82	Gamma	(24)
Diarrhoea	195.74	156.59	234.89	Gamma	(22)
Palmar-plantar erythrodysaesthesia syndrome	16.96	13.57	20.35	Gamma	(24)
Risk of AEs in toripalimab plus bevacizumab group					
Anaemia	6%	5%	7%	Beta	(12)
Hypertension	16%	13%	19%	Beta	(12)
Proteinuria	5%	4%	6%	Beta	(12)
Thrombocytopenia	10%	8%	12%	Beta	(12)
Aspartate aminotransferase increased	1%	0.80%	1.20%	Beta	(12)
Blood bilirubin increased	5%	4%	6%	Beta	(12)
Alanine aminotransferase increased	1%	0.80%	1.20%	Beta	(12)
Diarrhoea	1%	0.80%	1.20%	Beta	(12)
Palmar-plantar erythrodysaesthesia syndrome	0	NA	NA	NA	(12)
Risk of AEs in sorafenib group					
Anemia	4%	3%	5%	Beta	(12)
Hypertension	12%	10%	14%	Beta	(12)
Proteinuria	1%	0.80%	1.20%	Beta	(12)
Thrombocytopenia	3%	2%	4%	Beta	(12)
Aspartate aminotransferase increased	5%	4%	6%	Beta	(12)
Blood bilirubin increased	3%	2%	4%	Beta	(12)
Alanine aminotransferase increased	5%	4%	6%	Beta	(12)
Diarrhoea	7%	6%	8%	Beta	(12)
Palmar-plantar erythrodysaesthesia syndrome	10%	8%	12%	Beta	(12)
Proportion of subsequent systemic anti-cancer treatment					
Toripalimab plus bevacizumab group	56.80%	45.40%	68.20%	Beta	(12)
Sorafenib group	68.90%	55.10%	82.70%	Beta	(12)
Proportion of subsequent treatment in toripalimab plus bevacizumab group					
Targeted therapy	47.50%	38.00%	57.00%	Beta	(12)
Immunotherapy	27.20%	21.80%	32.60%	Beta	(12)
Best supportive care	43.20%	34.60%	51.80%	Beta	(12)
Proportion of subsequent treatment in sorafenib group					
Targeted therapy	57.90%	46.30%	69.50%	Beta	(12)
Immunotherapy	44.50%	35.60%	53.40%	Beta	(12)
Best supportive care	31.10%	24.90%	37.30%	Beta	(12)
Health utility inputs					
PFS	0.76	0.61	0.91	Beta	(22)
PD	0.68	0.54	0.82	Beta	(22)
Disutility inputs					
Anemia	0.073	0.058	0.088	Beta	(27)
Hypertension	0.016	0.013	0.019	Beta	(28)
Proteinuria	0.12	0.096	0.144	Beta	(29)
Thrombocytopenia	0.05	0.04	0.06	Beta	(30)
Aspartate aminotransferase increased	0	NA	NA	NA	(31)
Blood bilirubin increased	0	NA	NA	NA	(31)
Alanine aminotransferase increased	0	NA	NA	NA	(31)
Diarrhea	0.05	0.04	0.06	Beta	(31)
Palmar-plantar erythrodysaesthesia syndrome	0.15	0.12	0.18	Beta	(28)
Patient weight (kg)	65	52	78	Gamma	(19)
Discount rate (%)	5	0	8	Fixd	(15)

OS, overall survival; PFS, progression-free survival; PD, progressive disease; AE, adverse events; NA, not applicable.

### 2.2 Model construction

**Figure 1:**
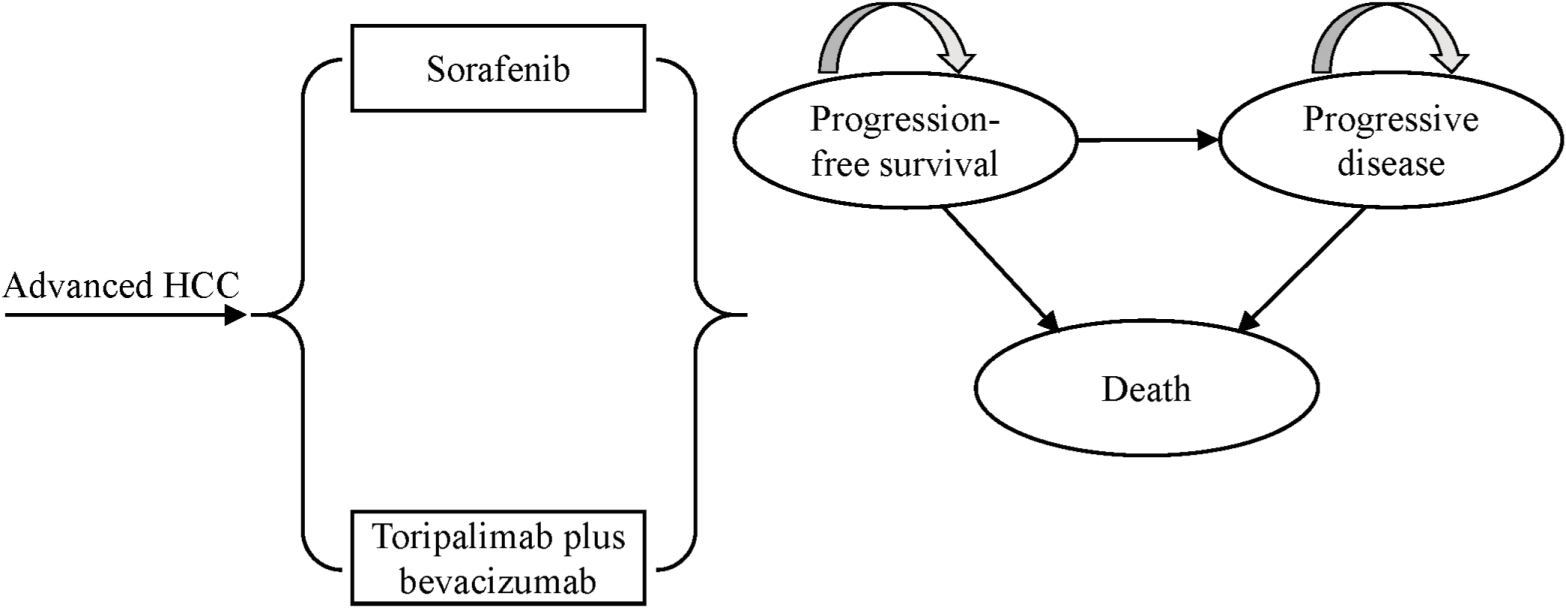
The structure of partitioned survival model for advanced HCC. HCC, hepatocellular carcinoma.

A retrospective analysis of National Institute for Health and Clinical Excellence (NICE) technology appraisals in oncology revealed that approximately 77% of 30 manufacturer submissions between 2013 and 2016 utilized partitioned survival models (14). In line with this methodological preference, we constructed a partitioned survival model using TreeAge Pro 2019 to evaluate the long-term cost-effectiveness of toripalimab plus bevacizumab versus sorafenib in patients with advanced HCC. The model comprised three mutually exclusive health states: PFS, progressive disease (PD), and death. All simulated patients entered the model in the PFS state. Over time, patients can transition from PFS to PD or death, and from PD to death, or remain in their current state, but disease progression is irreversible, meaning patients cannot return to the PFS state from the PD state (**Figure 1**). Health state occupancy proportion was directly estimated from the area under the corresponding survival curves: the proportion of surviving patients was derived from the area under the OS curve, while the mortality proportion was calculated as 1 minus the OS area. The PFS proportion was estimated from the area under the PFS curve, and the PD proportion was determined by the area between the OS and PFS curves. The model employed a 21-day cycle length to align with the treatment schedule in clinical trials, with a 10-year time horizon to ensure most patients entered the death state. Model outputs included total costs, life-years (LYs), quality-adjusted life-years (QALYs), and incremental cost-effectiveness ratios (ICERs). In accordance with the *China Guidelines for Pharmacoeconomic Evaluations 2020*, both costs and health outcomes were discounted at an annual rate of 5%. A willingness-to-pay (WTP) threshold of three times per capita gross domestic product (GDP) of China in 2024 ($40,334/QALY) was applied to judge the economic feasibility of the two treatment strategies. If the calculated ICER is below the WTP threshold, toripalimab plus bevacizumab is deemed cost-effective; otherwise, it is not considered economically favorable (15).

### 2.3 Clinical data

The clinical efficacy and safety data were derived from the HEPATORCH trial. Given the unavailability of original individual-level patient data and the need to extrapolate survival curves beyond the clinical trial follow-up period, standard parametric survival modeling was employed. The methodology proceeded as follows: First, PFS and OS data points were extracted from the published Kaplan-Meier (KM) curves in the HEPATORCH study using GetData Graph Digitizer software (version 2.26). Individual patient data (IPD) were then reconstructed using the algorithm proposed by Guyot et al. (16), implemented in R software (version 4.3.2). Seven parametric distributions were evaluated for survival curve fitting and extrapolation: Exponential, Weibull, Gompertz, Gamma, Generalized Gamma, Log-normal, and Log-logistic. Model selection was based on goodness-of-fit statistics using the Akaike Information Criterion (AIC), Bayesian Information Criterion (BIC), and visual inspection of clinical plausibility, with lower AIC and BIC values indicating better fit (17) (**Supplementary Table S3**; **Supplementary Figure S1**). To internally validate the reconstruction accuracy, the median survival estimates from the reconstructed curves were compared with those reported in the original trial (**Supplementary Table S2**). The Log-normal distribution provided the best fit for both treatment arms in the final analysis. The survival function is expressed as: $S\left(t\right)=1-\text{Φ}\left(\frac{\ln t-\text{μ}}{\text{σ}}\right)$, where Φ represents the standard normal cumulative distribution function. The estimated model parameters for survival distributions are presented in **Table 1**.

### 2.4 Costs estimate

This cost-effectiveness analysis (CEA) adopted a healthcare system perspective and only considered direct medical costs. The model incorporated the following cost categories: drug acquisition for first-line therapy, second-line treatment after disease progression, BSC, routine follow-up and monitoring, drug administration, management of adverse events (AEs), and end-of-life care. Drug pricing data were sourced from the latest 2025 average tender prices in the Yaozh database (https://data.yaozh.com/) (18). For drug cost calculations, we assumed a standard patient body weight of 65 kg (19). The drug cost per cycle was calculated as unit price multiplied by the total administered dose per cycle, without accounting for drug wastage. All other relevant cost parameters were derived from previously published literature (20–25). Only grade 3 or higher AE with incidence rates exceeding 5% in clinical trials were included in the model. The total AE-related treatment costs were estimated by multiplying the management cost per AE event by its corresponding incidence rate. All costs were adjusted to 2024 values using local Consumer Price Index (CPI) data through the inflation adjustment formula provided by https://www.inflationtool.com and subsequently converted to US dollars based on the 2024 average exchange rate ($1 = RMB 7.1217).

### 2.5 Health utilities estimate

This study employed health utility weights to quantify patient preferences for health outcomes associated with the interventions. Utility values ranging from 0 (death) to 1 (perfect health) were assigned to each health state. Following the approach of Durkee et al. (26), we assumed that health state utilities were independent of treatment regimen but influenced by treatment-related AEs and disease progression. Since health-related quality of life data were not reported in the HEPATORCH trial, utility values were derived from a published CEA study for advanced HCC, with utility of 0.76 for PFS and 0.68 for PD states (22). The disutility values associated with AEs were obtained from previously published studies (27–31). The total QALYs loss attributable to AEs was calculated by multiplying each AE disutility value by its corresponding incidence rate. All AEs were assumed to occur during the initial cycle of the model (23). Cost and utility parameters used in the model are summarized in **Table 1**.

### 2.6 Scenario analyses

To address potential uncertainties arising from modeling assumptions, this study conducted several scenario analyses: First, considering that the assumed time horizon in CEA may significantly influence the value assessment of treatment strategies, we varied the time horizon (5, 15, and 20 years) in the model to evaluate its impact on ICER; Second, the KM curves were fitted and extrapolated using suboptimal parametric distributions (**Supplementary Table S4**). Third, based on the EuroQol 5-Dimensional data collected during the IMbrave150 trial as reported by NICE (32), we reassigned the PFS utilities for the toripalimab plus bevacizumab group and sorafenib group to 0.78 and 0.77, respectively, with a PD utility of 0.74. Fourth, a biosimilar of bevacizumab ($139.85 per 100 mg) (33) was used to replace the originator drug. Finally, LYs were used as a measure of effectiveness to explore the uncertainty in model outputs.

### 2.7 Sensitivity analyses

The robustness of base-case results was assessed through one-way and probabilistic sensitivity analyses (PSA). In the one-way sensitivity analysis, input parameters were individually adjusted to their upper and lower bounds to identify key drivers of model outputs. For drug costs, the lowest and highest winning bid prices across different regions were used as the lower and upper limits of model inputs, respectively. The variation ranges for other parameters were determined based on the 95% CI or ±20% of the baseline value as reported in the literature. Following *China Guidelines for Pharmacoeconomic Evaluations 2020*, the discount rate was varied between 0% and 8% (15), with results presented in a tornado diagram.

For the PSA, Monte Carlo simulation was performed with 5,000 iterations, simultaneously sampling all analyzed variables from their predefined statistical distributions. Cost parameters were assigned gamma distribution, while utility values and probability parameters followed beta distribution. The standard errors required for distribution specification were estimated using 10% of baseline values. Results were presented as incremental cost-effectiveness scatterplots and cost-effectiveness acceptability curves (CEAC).

## 3 Results

### 3.1 Base-case results

The base-case analysis results are shown in **Table 1**, over a 10-year time horizon the toripalimab plus bevacizumab regimen incurred total costs of $44,994.43 while yielding 2.26 LYs and 1.57 QALYs. In comparison, sorafenib monotherapy resulted in total costs of $35,014.79, yielding 1.79 LYs and 1.16 QALYs. Compared to sorafenib, the toripalimab-bevacizumab combination provided an incremental gain of 0.41 QALYs at an additional cost of $9,979.63, resulting in an ICER of $24,602.67 per QALY gained. As this ICER is below the WTP threshold of 3 times China’s 2024 per capita GDP, toripalimab plus bevacizumab is considered a cost-effective treatment option for advanced HCC.

### 3.2 Scenario analyses results

The scenario analysis showed that the ICER increased or decreased when shortening or extending the time horizon. While using suboptimal distributions or considering LYs as health outcomes reduced the ICER values, the changes were not significant. In the analysis using alternative utility values, the ICER was $23,433.41/QALY. With the availability of the bevacizumab biosimilar, the ICER dropped to $20,520.73/QALY. Importantly, all estimated ICERs remained below the WTP threshold, robustly supporting the base-case results. The results of the scenario analyses are shown in **Table 2**.

**Table 2:** Summary of base-case and scenario analyses results.

Strategies and scenarios	Total cost ($)	LYs	QALYs	ICER ($/LY)	ICER ($/QALY)
Base-case analysis					
Sorafenib	35,014.79	1.79	1.16	NA	NA
Toripalimab plus bevacizumab	44,994.43	2.26	1.57	21,445.32	24,602.67
Scenario analysis (time horizon = 5 years)					
Sorafenib	33,394.81	1.62	1.05	NA	NA
Toripalimab plus bevacizumab	42,364.84	1.94	1.35	27,555.19	29,065.73
Scenario analysis (time horizon = 15 years)					
Sorafenib	35,401.00	1.84	1.19	NA	NA
Toripalimab plus bevacizumab	45,804.85	2.35	1.63	20,125.58	23,587.24
Scenario analysis (time horizon = 20 years)					
Sorafenib	35,542.28	1.85	1.20	NA	NA
Toripalimab plus bevacizumab	46,115.28	2.39	1.66	19,621.48	23,182.61
Scenario analysis (employing suboptimal distribution)					
Sorafenib	35,456.33	1.82	1.18	NA	NA
Toripalimab plus bevacizumab	44,655.20	2.28	1.59	20,075.68	22,698.75
Scenario analysis (varying utility value)					
Sorafenib	35,014.79	1.79	1.25	NA	NA
Toripalimab plus bevacizumab	44,994.43	2.26	1.67	21,445.32	23,433.41
Scenario analysis (bevacizumab biosimilar)					
Sorafenib	35,014.79	1.79	1.16	NA	NA
Toripalimab plus bevacizumab	43,338.66	2.26	1.57	17,887.23	20,520.73

LYs, life-years; QALYs, quality-adjusted life-years; ICER, incremental cost-effectiveness ratio; NA, not applicable.

### 3.3 Sensitivity analyses results

**Figure 2:**
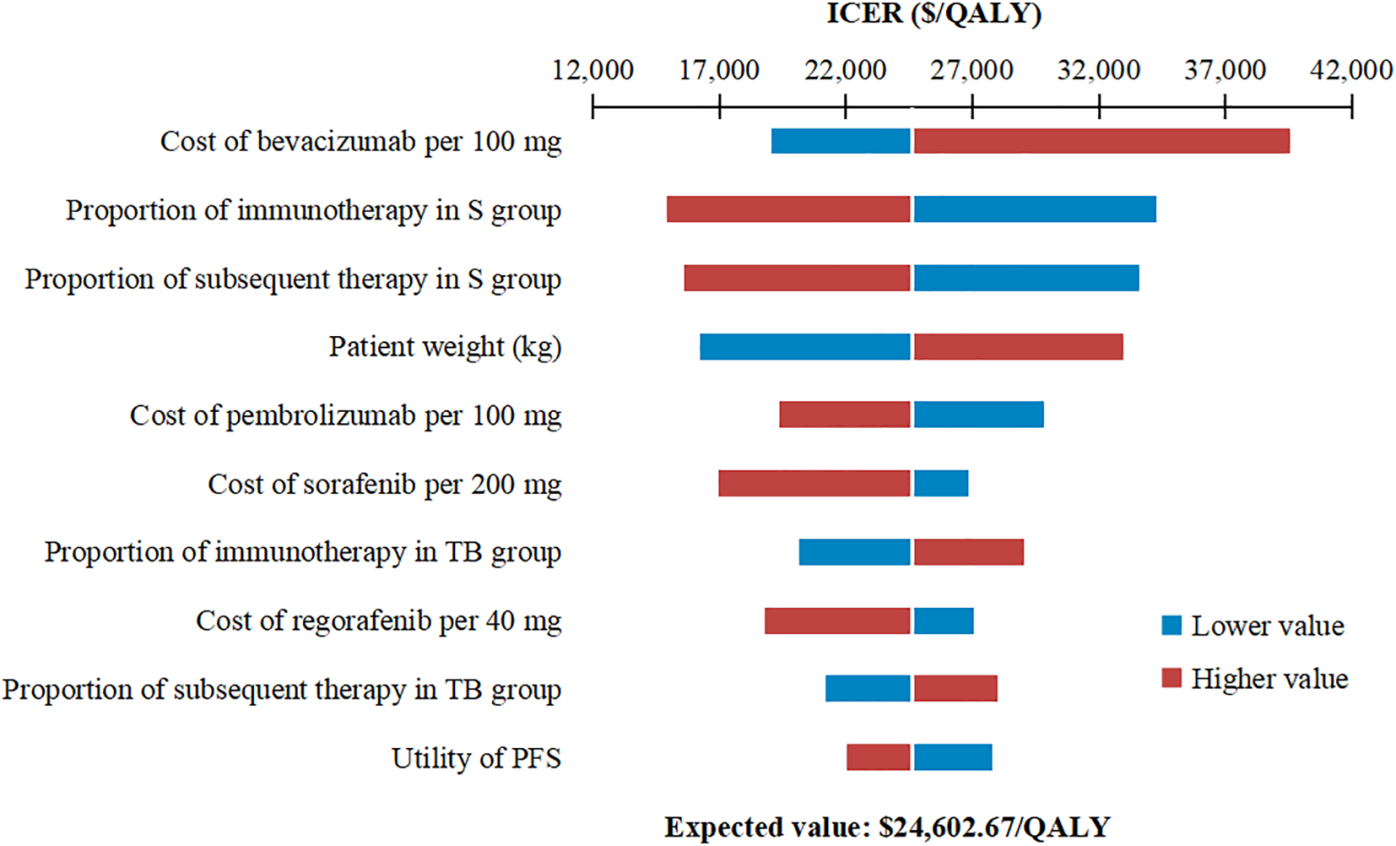
Tornado diagram of one-way sensitivity analysis of toripalimab plus bevacizumab versus sorafenib. ICER, incremental cost-effectiveness ratio; PFS, progression-free survival; S, sorafenib; TB, toripalimab plus bevacizumab.

**Figure 3:**
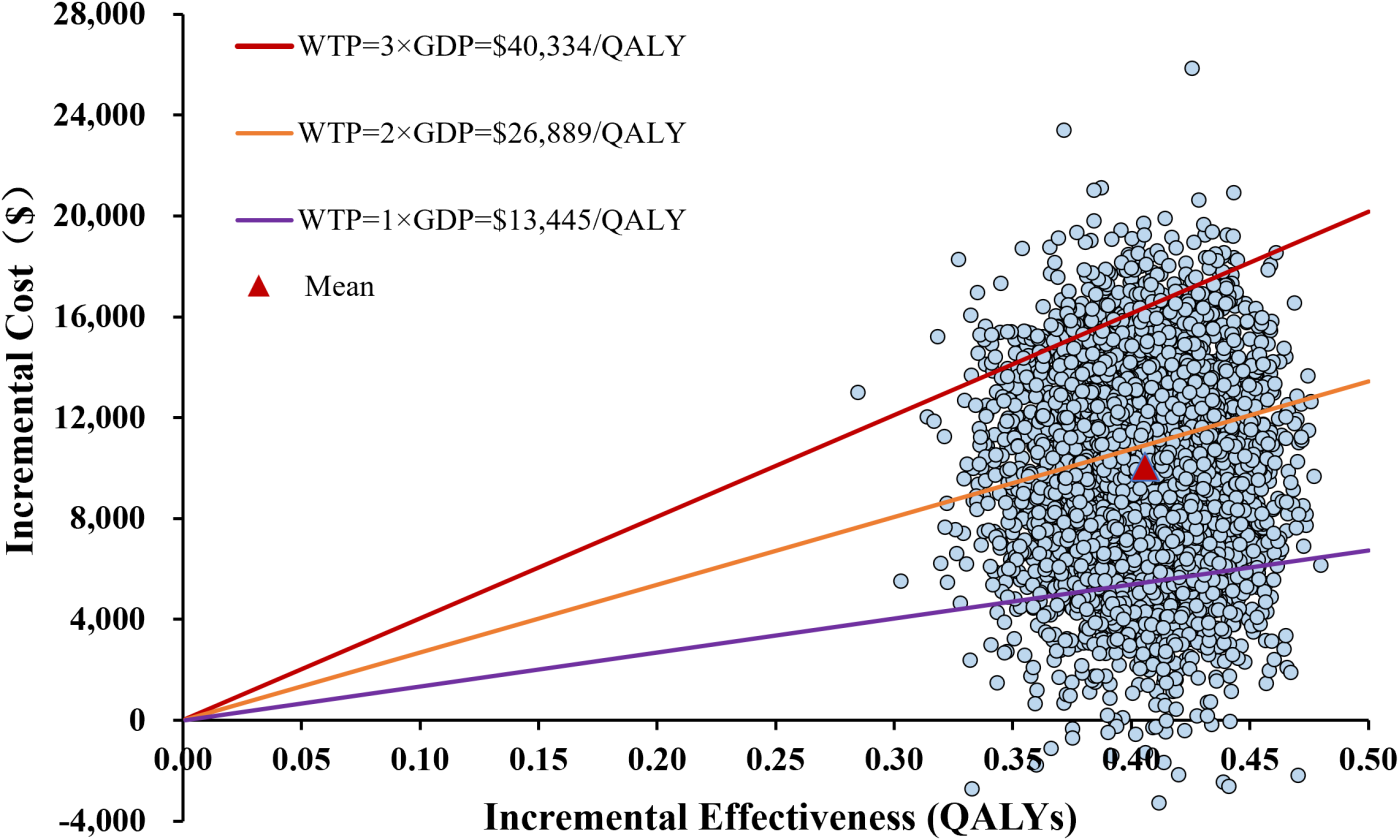
The incremental cost-effectiveness scatterplot in the probabilistic sensitivity analysis of toripalimab plus bevacizumab versus sorafenib. QALYs, quality-adjusted life-years; WTP, willingness-to-pay; ICER, incremental cost-effectiveness ratio; GDP, gross domestic product.

**Figure 4:**
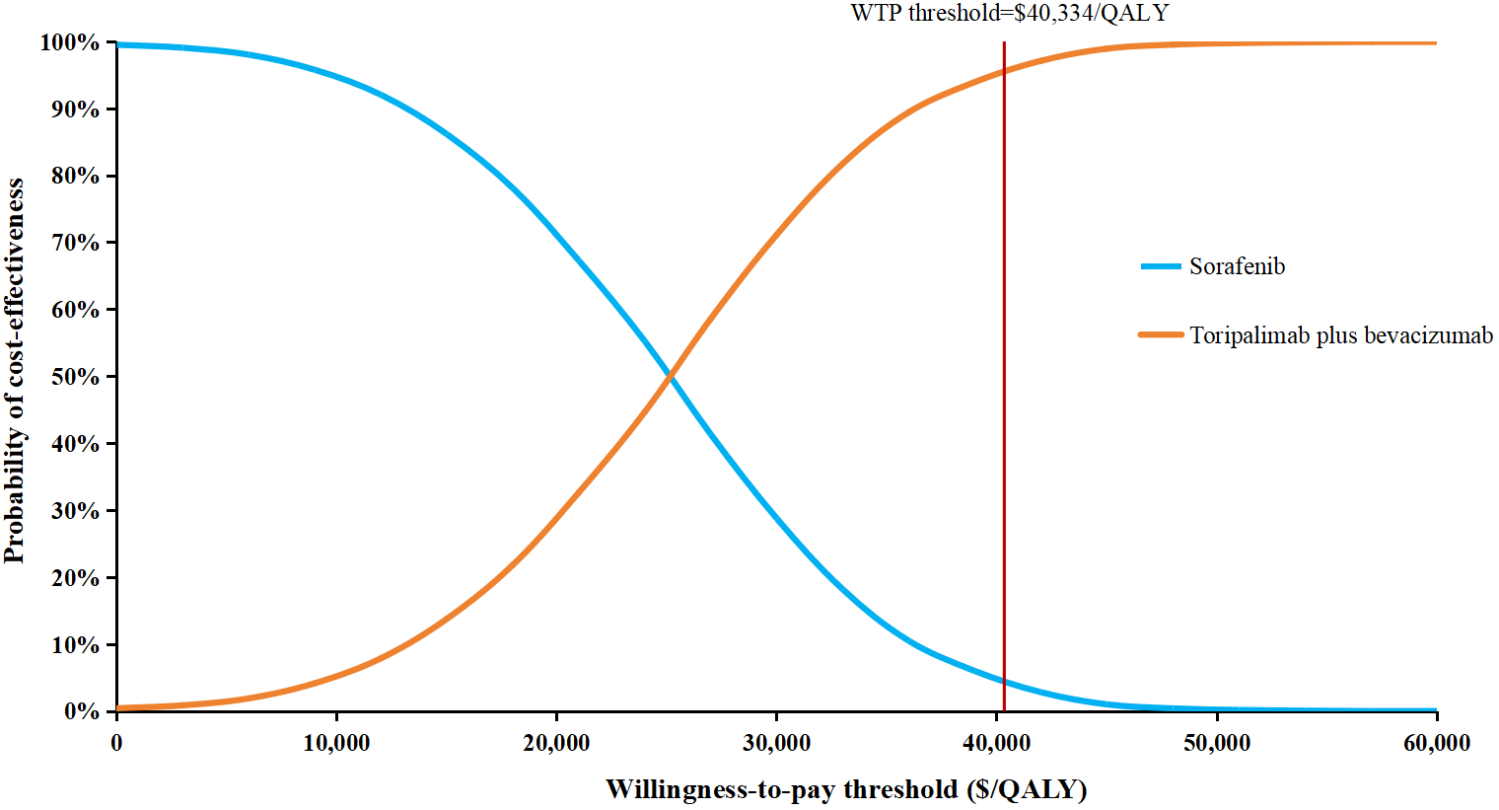
Cost-effectiveness acceptability curve of toripalimab plus bevacizumab compared to sorafenib. QALY, quality-adjusted life-year; WTP, willingness-to-pay.

The one-way sensitivity analysis, presented as a tornado diagram (**Figure 2**), identified the ten most influential parameters. The cost of bevacizumab, the proportion of patients receiving immunotherapy and subsequent therapy in the sorafenib group, and patient body weight greatly influenced the ICER. Specifically, all ICERs ranged from $14,938.13 to $39,563.51 per QALY. Nevertheless, throughout all tested parameter ranges, ICER consistently remained below the WTP threshold without reversing conclusion. For the PSA, the incremental cost-effectiveness scatter plot showed most scatter points located in the first quadrant (more effective and more costly) (**Figure 3**). The mean ICER from the probabilistic sampling was $24,816.12/QALY, which is close to the ICER obtained in the base-case analysis. At WTP thresholds of $13,445 (1×GDP per capita), $26,889 (2×GDP), and $40,334 (3×GDP) per QALY, the proportions of scatter points falling below the threshold lines were 10.50%, 58.16%, and 95.76%, respectively. The CEAC illustrated the probability of each strategy being cost-effective across a range of WTP thresholds (**Figure 4**). When the WTP threshold reached approximately $25,168 per QALY, the combination of toripalimab and bevacizumab achieved a probability of cost-effectiveness greater than 50%. At the WTP threshold of $40,334 per QALY, the cost-effectiveness acceptability of this combination regimen was 95.76%.

## 4 Discussion

HCC represents a significant global health challenge. Sorafenib, as the conventional first-line treatment for advanced HCC, has demonstrated limited clinical efficacy in recent years. With elucidation of the tumor immune microenvironment mechanisms, ICIs have profoundly transformed the HCC treatment paradigm, offering patients with more effective therapeutic options (34). Currently, the combination of ICIs with anti-angiogenic targeted therapy has emerged as a crucial first-line treatment for advanced HCC. The HEPATORCH study, presented at the 2024 Chinese Society of Clinical Oncology (CSCO) Annual Meeting, reported groundbreaking results for toripalimab combination therapy in first-line advanced HCC treatment. This regimen has demonstrated substantial improvements in clinical outcomes, not only renewing hope for HCC immunotherapy but also establishing robust evidence-based foundation for the immunology-antiangiogenesis combination strategy.

Our study demonstrated that compared with sorafenib monotherapy, the incremental cost per QALY gained with toripalimab-bevacizumab combination was $24,602.67. The ICER value was substantially lower than the cost-effectiveness threshold of three times China’s per capita GDP in 2024. Therefore, toripalimab plus bevacizumab was regarded as a cost-effective treatment strategy. Notably, even when the WTP threshold was reduced to 2 times GDP per capita, the combination of toripalimab and bevacizumab still exhibited an economic feasibility of 58.16%. When the WTP threshold approached $25,168/QALY, there was a greater than 50% chance of being cost-effective. This further suggested the superior cost-effectiveness of the toripalimab combined with bevacizumab.

Although immunotherapy incurs higher initial costs, its mechanism of action typically leads to delayed treatment effects and prolonged survival benefits in responding patients, resulting in gradual accumulation of QALYs over time (35, 36). In this economic evaluation model, aligning with previous studies, we set the time horizon for the base-case at 10 years and validated the results using both short-term and long-term scenarios. Therefore, varying time frames as an exploratory analysis is an effective method to capture all potential consequences. The results consistently support our primary conclusion across various time horizons. Furthermore, in the scenario with biosimilar bevacizumab available, its use reduces the total cost of the toripalimab combined with bevacizumab regimen, improving its cost-effectiveness. The finding is similar to the economic evaluation results of the atezolizumab-bevacizumab combination by Chang et al. (37) Moreover, the tornado diagram identified the cost of bevacizumab as the most sensitive parameter affecting the ICER, suggesting that reducing its cost would further enhance the economic advantage of the toripalimab plus bevacizumab regimen. Based on these results, the introduction of bevacizumab biosimilars into treatment combinations holds considerable value as it maintains clinical efficacy while reducing healthcare expenditures and improving treatment accessibility. Additionally, market entry of biosimilars will further optimize drug pricing systems through competition mechanisms.

The standard for diagnosis and treatment of primary liver cancer (2024 edition) (38) lists apatinib, regorafenib, ramucirumab, pembrolizumab, camrelizumab, and tislelizumab as first-line recommended strategies for second-line treatment of advanced HCC. Based on the results of the RESORCE (39) and KEYNOTE-240 trials (40), regorafenib and pembrolizumab were selected as the preferred targeted and immunotherapy drugs for second-line treatment in this study, respectively. The one-way sensitivity analysis indicated that the proportion of patients receiving immunotherapy and the proportion receiving subsequent therapy in the sorafenib group ranked second and third, respectively, in terms of their influence on model outcomes. As the proportion of patients in the sorafenib group receiving second-line therapy increased, the ICER gradually decreased. The result was primarily driven by a higher disease progression proportion in the sorafenib group compared to the toripalimab-bevacizumab group, along with a greater proportion receiving second-line therapy (68.9% vs. 56.8%), thereby reducing the incremental cost between the two treatment strategies. Fortunately, this did not lead to a reversal in the economic outcomes of the two strategies.

Several relevant studies warrant discussion. Gong et al. (8) conducted a systematic review of economic evaluations of ICIs as a treatment for advanced HCC from 2010 to 2024. The results indicated that, compared with sorafenib, the combinations of atezolizumab with bevacizumab and sintilimab with bevacizumab or its biosimilars were not cost-effective for first-line treatment of advanced HCC in China. Later, Liu et al. (19) reassessed the atezolizumab-bevacizumab regimen with updated data and affirmed its lack of cost-effectiveness. Chang et al. (37) based on the healthcare payment system in Taiwan, China, pointed out that the cost of atezolizumab combined with bevacizumab would need to be reduced to 70% of the original price to achieve cost-effectiveness. It is evident that drug pricing is a key factor leading to unfavorable cost-effectiveness outcomes. In contrast, Cai et al. (41) and Zhao et al. (24) reported that the combination of camrelizumab and rivoceranib as a viable first-line treatment option for advanced HCC in China has considerable cost-effectiveness. The aforementioned studies share similarities with the current study in terms of treatment approaches, all involving the combination of ICIs with anti-angiogenic targeted therapy. This combined strategy, although enhancing efficacy through the synergistic effects of immune modulation and angiogenesis inhibition, faces the high drug pricing as the primary economic barrier to large-scale clinical adoption. Compared with other ICIs, toripalimab, as the first domestically developed and approved PD-1 inhibitor in China, has a more competitive pricing and lower transportation costs due to localized production, thus potentially offering broader accessibility and application among Chinese patients (42).

The strengths of this study are noteworthy and deserve emphasis. To our knowledge, this is the first to assess the cost-effectiveness of first-line toripalimab plus bevacizumab therapy for patients with advanced HCC from the perspective of China’s healthcare system using economic modeling. Although several PD-1/PD-L1 and antiangiogenic combinations have been approved as first-line treatments, unmet needs persist due to the high morbidity and mortality of HCC, along with regional approval status, reimbursement restrictions, and cost considerations (12). While previous economic evaluations have primarily focused on other PD-1/PD-L1 inhibitor-based antiangiogenic therapies, our analysis specifically investigates this unique domestically developed PD-1 inhibitor regimen by integrating most recent clinical evidence and ensuring robustness through scenario and sensitivity analyses. These findings offer timely and regionally relevant evidence to inform medical decision-making for advanced HCC treatment in China.

This study has some limitations that should be acknowledged. First, the use of survival models to fit and extrapolate KM curves may introduce potential errors, particularly during long-term extrapolation where overestimation or underestimation of survival benefits could increase uncertainty in the economic evaluation. Second, the lack of utility values from the HEPATORCH study necessitated the use of external data, which may not accurately reflect patient quality of life, though scenario analyses confirmed the consistency of base-case results. Future research on localized quality-of-life studies is urgently needed to provide reliable health utility data for pharmacoeconomic evaluations. Third, only grade three or higher AEs in the first cycle were included, potentially underestimating costs and overestimating QALYs, although sensitivity analysis showed these parameters had slight impact on model outcomes. Fourth, limited details on second-line treatment in the HEPATORCH trial required assumptions about subsequent anticancer therapies, which may not fully align with real-world clinical practice. Fifth, the absence of head-to-head clinical trials restricted the comparison to sorafenib alone, excluding other first-line ICI combination regimens. Lastly, considering that the subjects in the study were mainly from mainland China, the study results should be cautiously extrapolated to a broader population.

## 5 Conclusion

In summary, from the China’s healthcare system perspective and at current pricing and commonly accepted WTP threshold, toripalimab plus bevacizumab may represent a cost-effective first-line option for advanced HCC compared with sorafenib. This economic evidence provides valuable support for clinical decision-making, although future validation with real-world data will be essential to substantiate these findings.

## Data Availability

The original contributions presented in the study are included in the article/**Supplementary Material**. Further inquiries can be directed to the corresponding author.
