# Supplementary material for: Cost-effectiveness analysis of toripalimab plus bevacizumab versus sorafenib as first-line treatment for advanced hepatocellular carcinoma in China
Source: Front Immunol. 2025 Dec 9;16:1676293. doi: 10.3389/fimmu.2025.1676293 (PMC12722981; doi:10.3389/fimmu.2025.1676293)
Supplement: Supplementary file 1 [file DataSheet1.pdf]

# Cost-effectiveness analysis of toripalimab plus bevacizumab versus sorafenib as first-line treatment for advanced hepatocellular carcinoma in China

**Zhengxiong Li[1], Jing He[2], Kai Ma[1*]**

[1]School of Medical Informatics and Engineering, Xuzhou Medical University, Xuzhou, China

[2]School of Medicine, University of Electronic Science and Technology of China, Chengdu, China

*** Correspondence:**

Kai Ma

cumtbmakai@126.com

# *Supplementary Material*

**Supplementary Table S1** | CHEERS Checklist 2022.

**Supplementary Table S2** | The median survival of original and reconstructed Kaplan-Meier survival curves.

**Supplementary Table S3** | Summary of statistical goodness-of-fit of Kaplan-Meier curves.

**Supplementary Table S4** | The survival model parameters for suboptimal distributions of survival curves.

**Supplementary Figure S1** | Fitting and extrapolation of Kaplan-Meier survival curves.

## 1 Supplementary Tables

**Supplementary Table S1.** CHEERS Checklist 2022.

| Topic | No | Item | Reported |
|---|---|---|---|
| **Title** | | | |
| Title | 1 | Identify the study as an economic evaluation and specify the interventions being compared | Yes |
| **Abstract** | | | |
| Abstract | 2 | Provide a structured summary that highlights context, key methods, results, and alternative analyses | Yes |
| **Introduction** | | | |
| Background and objectives | 3 | Give the context for the study, the study question, and its practical relevance for decision making in policy or practice | Yes |
| **Methods** | | | |
| Health economic analysis plan | 4 | Indicate whether a health economic analysis plan was developed and where available | Not applicable |
| Study population | 5 | Describe characteristics of the study population (such as age range, demographics, socioeconomic, or clinical characteristics) | Yes |
| Setting and location | 6 | Provide relevant contextual information that may influence findings | Yes |
| Comparators | 7 | Describe the interventions or strategies being compared and why chosen | Yes |
| Perspective | 8 | State the perspective(s) adopted by the study and why chosen | Yes |
| Time horizon | 9 | State the time horizon for the study and why appropriate | Yes |
| Discount rate | 10 | Report the discount rate(s) and reason chosen | Yes |
| Selection of outcomes | 11 | Describe what outcomes were used as the measure(s) of benefit(s) and harm(s) | Yes |
| Measurement of outcomes | 12 | Describe how outcomes used to capture benefit(s) and harm(s) were measured | Yes |
| Valuation of outcomes | 13 | Describe the population and methods used to measure and value outcomes | Yes |
| Measurement and valuation of resources and costs | 14 | Describe how costs were valued | Yes |
| Currency, price date, and conversion | 15 | Report the dates of the estimated resource quantities and unit costs, plus the currency and year of conversion | Yes |
| Rationale and description of model | 16 | If modelling is used, describe in detail and why used. Report if the model is publicly available and where it can be accessed | Yes |
| Analytics and assumptions | 17 | Describe any methods for analysing or statistically transforming data, any extrapolation methods, and approaches for validating any model used | Yes |

| | | | |
|---|---|---|---|
| Characterising heterogeneity | 18 | Describe any methods used for estimating how the results of the study vary for subgroups | Not applicable |
| Characterising distributional effects | 19 | Describe how impacts are distributed across different individuals or adjustments made to reflect priority populations | Not applicable |
| Characterising uncertainty | 20 | Describe methods to characterise any sources of uncertainty in the analysis | Yes |
| Approach to engagement with patients and others affected by the study | 21 | Describe any approaches to engage patients or service recipients, the general public, communities, or stakeholders (such as clinicians or payers) in the design of the study | Not applicable |
| **Results** | | | |
| Study parameters | 22 | Report all analytic inputs (such as values, ranges, references) including uncertainty or distributional assumptions | Yes |
| Summary of main results | 23 | Report the mean values for the main categories of costs and outcomes of interest and summarise them in the most appropriate overall measure | Yes |
| Effect of uncertainty | 24 | Describe how uncertainty about analytic judgments, inputs, or projections affect findings. Report the effect of choice of discount rate and time horizon, if applicable | Yes |
| Effect of engagement with patients and others affected by the study | 25 | Report on any difference patient/service recipient, general public, community, or stakeholder involvement made to the approach or findings of the study | Not applicable |
| **Discussion** | | | |
| Study findings, limitations, generalisability, and current knowledge | 26 | Report key findings, limitations, ethical or equity considerations not captured, and how these could affect patients, policy, or practice | Yes |
| Other relevant information Source of funding | 27 | Describe how the study was funded and any role of the funder in the identification, design, conduct, and reporting of the analysis | Yes |
| Conflicts of interest | 28 | Report authors conflicts of interest according to journal or International Committee of Medical Journal Editors requirements | Yes |

**Supplementary Table S2.** The median survival of original and reconstructed Kaplan Meier survival curves.

| Group | mPFS (95% CI) | mOS (95% CI) |
|---|---|---|
| Toripalimab plus bevacizumab group-original | 5.8 (4.6, 7.2) | 20.0 (15.3, 23.4) |
| Toripalimab plus bevacizumab group-reconstructed | 5.5 (4.3, 7.1) | 20.1 (16.4, 25.0) |
| Sorafenib group-original | 4.0 (2.8, 4.2) | 14.5 (11.4, 18.8) |
| Sorafenib group-reconstructed | 4.0 (2.8, 4.4) | 14.5 (11.7, 19.8) |

Abbreviation: mPFS, median progression-free survival; mOS, median overall survival.

**Supplementary Table S3.** Summary of the statistical goodness-of-fit of Kaplan-Meier survival curves.

| Parameters | Exponential | Weibull | Gamma | Generalized gamma | Gompertz | Log-normal | Log-logistic |
|---|---|---|---|---|---|---|---|
| Toripalimab + bevacizumab-overall survival curve | | | | | | | |
| AIC | 900.653176 | 900.481373 | 898.942193 | 883.314707 | 902.653148 | 887.200466 | 893.613965 |
| BIC | 903.740772 | 906.656565 | 905.117385 | 892.577496 | 908.82834 | 893.375659 | 899.789158 |
| Sorafenib-overall survival curve | | | | | | | |
| AIC | 1048.56147 | 1045.36398 | 1043.90306 | 1042.58943 | 1048.87144 | 1041.39017 | 1042.54046 |
| BIC | 1051.66133 | 1051.56371 | 1050.1028 | 1051.88903 | 1055.07118 | 1047.5899 | 1048.74019 |
| Toripalimab + bevacizumab-progression-free survival curve | | | | | | | |
| AIC | 667.865828 | 668.320203 | 667.100443 | 647.738398 | 669.790211 | 654.982028 | 663.521442 |
| BIC | 670.953424 | 674.495396 | 673.275636 | 657.001187 | 675.965404 | 661.157221 | 669.696635 |
| Sorafenib-progression-free survival curve | | | | | | | |
| AIC | 610.444279 | 604.981408 | 598.989503 | 554.218201 | 612.443045 | 576.360746 | 583.024083 |
| BIC | 613.544145 | 611.181141 | 605.189236 | 563.5178 | 618.642778 | 582.560479 | 589.223816 |

**Supplementary Table S4.** The survival model parameters for suboptimal distributions of survival curves.

| Group | Suboptimal distribution | Key parameters |
|---|---|---|
| Toripalimab plus bevacizumab group-OS | Log-logistic | Shape =1.451, scale = 18.776 |
| Toripalimab plus bevacizumab group-PFS | Log-logistic | Shape = 1.429, scale = 5.296 |
| Sorafenib group-OS | Log-logistic | Shape = 1.595, scale = 14.791 |
| Sorafenib group-PFS | Log-logistic | Shape = 1.841, scale = 3.706 |

Abbreviation: PFS, progression-free survival; OS, overall survival.

## 2 Supplementary Figures

A

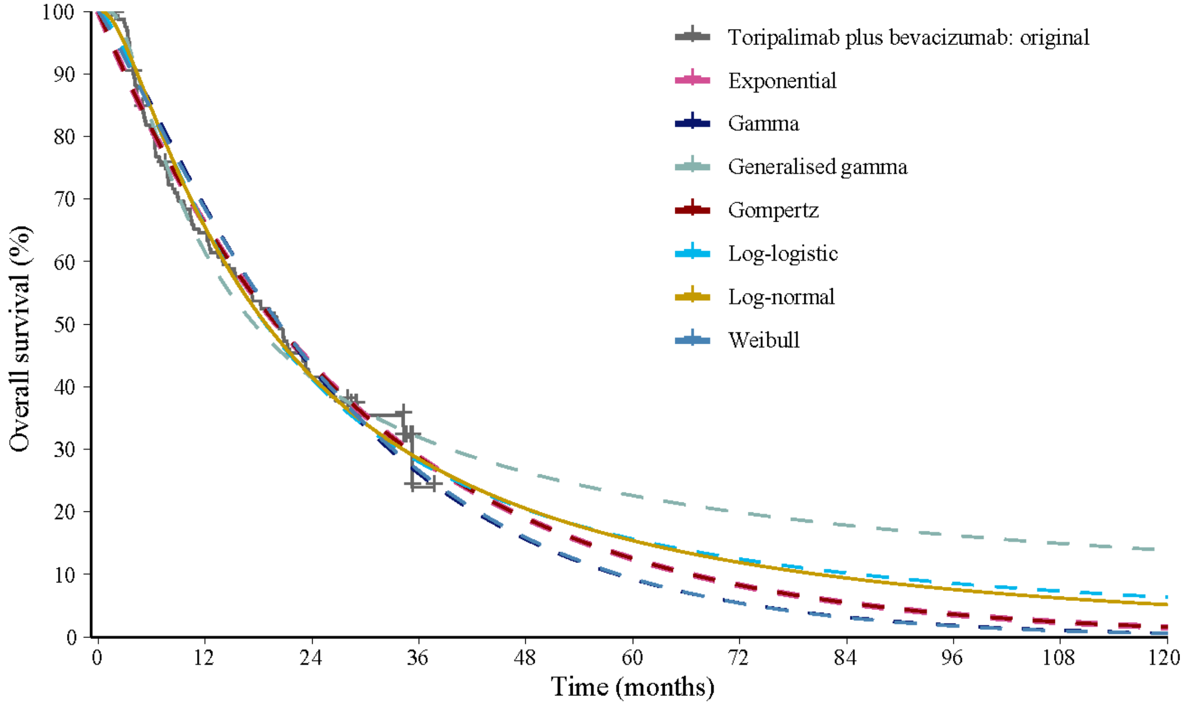


B

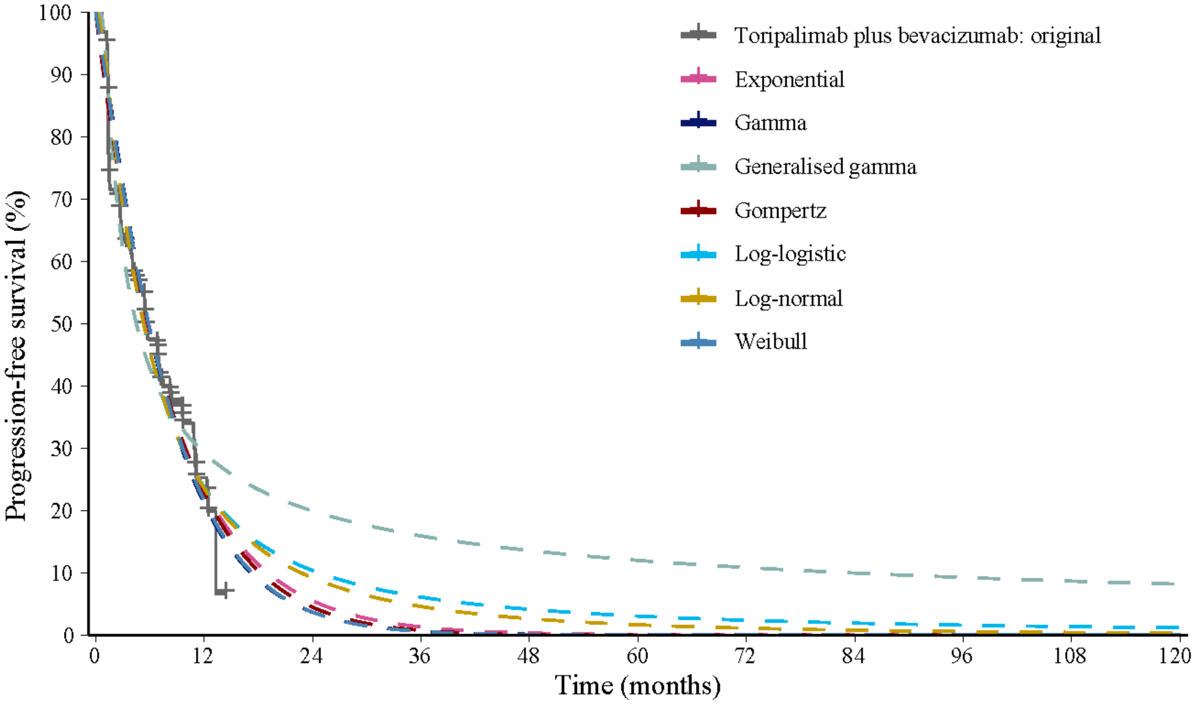

C

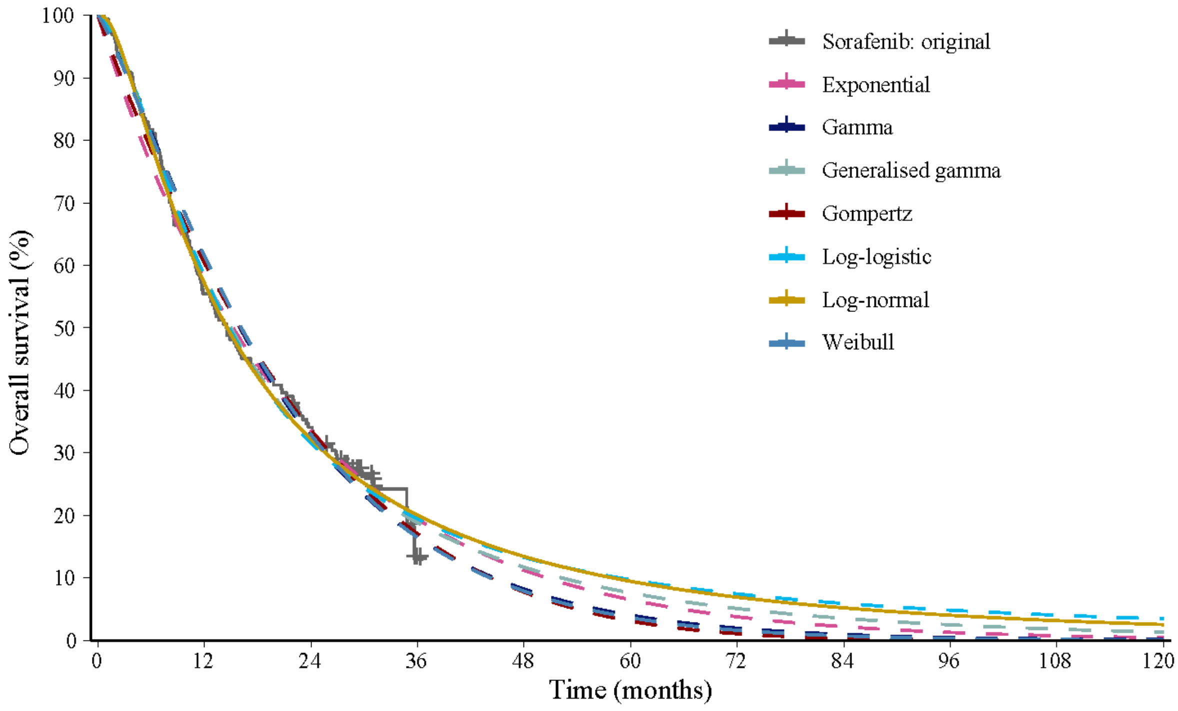


D

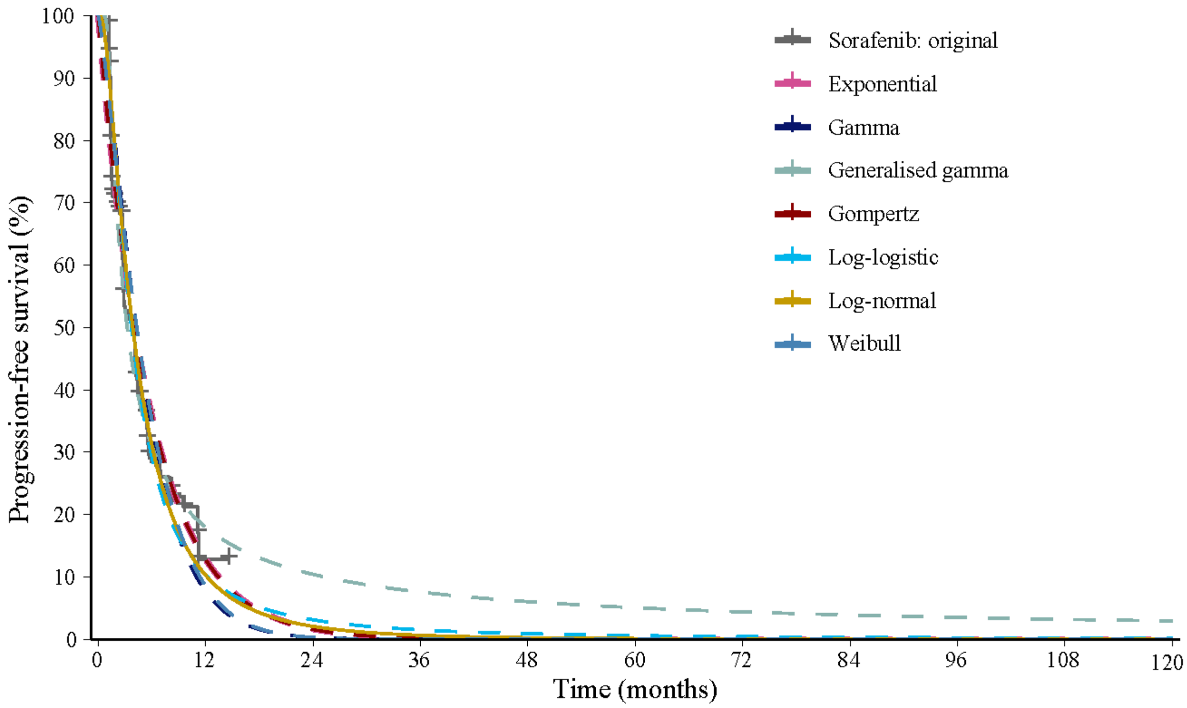


**Supplementary Figure S1.** Fitting and extrapolation of Kaplan-Meier survival curve.
